# Salicylic acid mediated immune response of *Citrus sinensis* to varying frequencies of herbivory and pathogen inoculation

**DOI:** 10.1186/s12870-021-03389-5

**Published:** 2022-01-03

**Authors:** Freddy Ibanez, Joon Hyuk Suh, Yu Wang, Monique Rivera, Mamoudou Setamou, Lukasz L. Stelinski

**Affiliations:** 1 Department of Entomology and Nematology, Citrus Research and Education Center, University of Florida, Lake Alfred, Florida, 33850 USA; 2 Present address: Texas A&M University-AgriLife Research, 2415 E Highway 83 –, Weslaco, TX 78596 USA; 3 Department of Food Science and Human Nutrition, Citrus Research and Education Center, University of Florida, Lake Alfred, Florida, 33850 USA; 4 Department of Entomology, University of California Riverside, Riverside, California, 92521 USA; 5 Texas A&M University-Kingsville Citrus Center, 312 N International Blvd, Weslaco, TX 78599 USA

**Keywords:** Salicylic acid, *Diaphorina citri*, Plant defense, Metabolomics, Gene expression, Vector-host-pathogen interactions, Huanglongbing

## Abstract

**Background:**

Plant immunity against pathogens and pests is comprised of complex mechanisms orchestrated by signaling pathways regulated by plant hormones [Salicylic acid (SA) and Jasmonic acid (JA)]. Investigations of plant immune response to phytopathogens and phloem-feeders have revealed that SA plays a critical role in reprogramming of the activity and/or localization of transcriptional regulators via post-translational modifications. We explored the contributing effects of herbivory by a phytopathogen vector [Asian citrus psyllid, *Diaphorina citri*] and pathogen [*Candidatus* Liberibacter asiaticus (*Ca*Las)] infection on response of sweet orange [*Citrus sinensis* (L.) Osbeck] using manipulative treatments designed to mimic the types of infestations/infections that citrus growers experience when cultivating citrus in the face of Huanglongbing (HLB) disease.

**Results:**

A one-time (7 days) inoculation access period with *Ca*Las-infected vectors caused SA-associated upregulation of *PR-1*, stimulating defense response after a long period of infection without herbivory (270 and 360 days). In contrast, while repeated (monthly) ‘pulses’ of 7 day feeding injury by psyllids stimulated immunity in *Ca*Las-infected citrus by increasing SA in leaves initially (up to 120 days), long-term (270 and 360 days) repeated herbivory caused SA to decrease coincident with upregulation of genes associated with SA metabolism (*BMST* and *DMR6*). Similarly, transcriptional responses and metabolite (SA and its analytes) accumulation in citrus leaves exposed to a continuously reproducing population of *D. citri* exhibited a transitory upregulation of genes associated with SA signaling at 120 days and a posterior downregulation after long-term psyllid (adults and nymphs) feeding (270 and 360 days).

**Conclusions:**

Herbivory played an important role in regulation of SA accumulation in mature leaves of *C. sinensis*, whether or not those trees were coincidentally infected with *Ca*Las. Our results indicate that prevention of feeding injury inflicted by *D. citri* from the tritrophic interaction may allow citrus plants to better cope with the consequences of *Ca*Las infection, highlighting the importance of vector suppression as a component of managing this cosmopolitan disease.

**Supplementary Information:**

The online version contains supplementary material available at 10.1186/s12870-021-03389-5.

## Background

Huanglongbing (HLB), also known as citrus greening is a disease that limits production of commercially important *Citrus* spp. worldwide [1]. Three taxonomic species of pathogenic bacteria are associated with HLB: *Candidatus* Liberibacter asiaticus, Ca. Liberibacter africanus, and Ca. Liberibacter americanus. ‘*Ca. L. asiaticus*’ and ‘*Ca. L. americanus*’ are both transmitted by *Diaphorina citri* Kuwayama in Asia and the Americas [1] whereas ‘*Ca. L. africanus*’ is transmitted by the African citrus psyllid, *Trioza erytreae* Del Guercio (Hemiptera: Triozidae) on the African continent [1]. In Florida, only Ca. Liberibacter asiaticus (*Ca*Las) has been reported and is transmitted by the vector, Asian citrus psyllid, *D. citri* [1, 2]. Despite efforts to control the disease and/or vector, current strategies have had limited effect on curtailing disease spread resulting in lost commercial feasibility. Florida’s citrus industry has been devastated losing over $7 billion [3], putting the future of America’s citrus production capabilities at risk. Nevertheless, significant research effort targeting this problem is beginning to yield promising results, including identification of two varieties that exhibit levels of tolerance to *Ca*Las infection - ‘Bearss’ lemon, *Citrus limon* Burm. f., and ‘LB8–9’ Sugar Belle® mandarin hybrid (“Clementine” mandarin × “Minneola” tangelo). Lower levels of phloem disruption and greater phloem regeneration have been documented in these two varieties following *Ca*Las infection as compared with HLB-susceptible sweet orange, *Citrus sinensis* (L.) Osbeck [4]. However, the molecular mechanisms involved in this tolerance are still not understood and the biological events involved in immune defense systems of *Citrus* varieties require further investigation.

Plant defense mechanisms are an adaptive response to combat injurious organisms. Two innate immunity strategies *in planta* have evolved to detect pathogens and their mechanisms have been extensively reviewed [5–7]. The first strategy (barrier) is known as microbe-associated molecular patterns (MAMPs) or pathogen-associated molecular patterns (PAMPs) [8–10] wherein pattern-recognition receptors localized in the host-cell membrane interact with conserved molecules of different groups of microbes, including: lipids, carbohydrates, proteins, and other small molecules. This recognition activates PAMP-triggered immunity (PTI) leading to an array of defense responses [5]. The second strategy, effector-triggered immunity (ETI), involves the secretion of pathogen effectors that interplay with host proteins and are encoded by so-called resistance (R) genes. Successful pathogens that suppress host PTI responses, for instance the hypersensitive response, secrete specific effectors that modulate programmed cell death activated during PTI [11]. Most R genes encode nucleotide-binding site leucine-rich repeat (NB-LRR) proteins [12, 13]; for example, BRASSINOSTEROID INSENSITIVE 1-ASSOCIATED KINASE 1 (BAK1), which is targeted by bacterial effectors to interrupt the PTI response [14–16]. Recognition of bacterial molecules by PAMP or MAMP pattern-recognition receptors induces an oxidative burst produced by NADPH oxidase encoded by *rbohD* [17]. Also, such recognition activates the salicylic acid (SA)-dependent signaling pathway [18].

Plants not only must defend themselves against potential pathogens, but also encounter numerous herbivores during their lifetime that modulate specific biochemical and molecular responses, which vary depending on the type of feeding and/or oviposition occurring on the plant [19]. Investigations of plant-insect interactions using aphids and whiteflies as models have described induction of the SA-dependent immune response in plants as reviewed by Walling [20] and references therein. Similar investigations focusing on psyllids have demonstrated congruent immune responses in plants [21–23].

Plants synthetize SA using isochorismate synthase and phenylalanine ammonia-lyase enzymes and their respective pathways; these processes are reviewed in Chen et al. [24] and D’Maris Amick Dempsey et al. [25] and references therein. Recently, a non-enzymatic reaction for SA accumulation was described in *Arabidopsis thaliana*, wherein avrPphB Susceptible 3 (PBS3) in the cytosol catalyzes the formation of Isochorismate-9-glutamate (ISC-9-Glu), which later spontaneously decays into SA [26]. Afterward, SA is enzymatically modified by glycosylation, methylation, amino acid conjugation, and hydroxylation [25, 27]. The function(s) of SA metabolites have not been completely elucidated, but putative roles were ascribed in defense regulation [28].

The transcription cofactor, Nonexpresser of Pathogenesis Related genes 1 (NPR1), is a critical protein in plant defense during SA-dependent signaling. After nuclear translocation, NPR1 cofactor interacts with TGA transcription factors [29], and is proposed to function as a co-activator of gene expression for systemic acquired resistance (SAR) [30, 31]. Within the SA signaling pathway, two NPR1 paralogues, NPR3 and NPR4, were initially shown to interact with NPR1 and function as E3 ligases that degrade NPR1 in a SA concentration-regulated manner to activate gene expression associated with defense [32, 33]. However, a recent study in Arabidopsis showed that NPR3/NPR4 interacts with TGAs to inhibit defense-related gene expression when SA accumulation is low [26, 34]. During pathogen infection, SA increases and binds to NPR3/NPR4, which releases the transcriptional repression of defense genes, allowing SA to bind NPR1, which in turn, activates the transcription of defense genes. In a previous study, the mechanism(s) of immune response in *C. sinensis* via accumulation of SA and its metabolites was examined after 7-, 14-, and 150-days of feeding by uninfected *D. citri*. Depending on the duration of herbivory, the vector (*Ca*Las-free) differentially regulates transcription and accumulation of SA and its metabolites in mature leaves [23]. Overall, an increasing body of evidence suggests that immune responses against phloem feeders and pathogens are tightly regulated by SA accumulation. We hypothesized that the accumulation of SA and its metabolites are critical in *C. sinensis* to regulate the transcriptional response of genes involved in host-immune defenses in response to biotic stressors (pathogen and/or vector).

The overall goal of this study was to investigate modulation of SA-dependent plant defense responses in *C. sinensis* L. Osbeck (sweet orange), by exposing plants to three distinct frequencies and intervals of *D. citri* herbivory and *Ca*Las-inoculation access periods as manipulated stressors. Our results describe the molecular and SA-dependent metabolic events that occur during HLB disease progression and after various intervals of insect feeding and following various frequencies of *Ca*Las inoculation. Specifically, we analyzed expression of a selected group of genes involved in: SA-dependent immune response, and SA chemical modification. Concurrently, LC/MS analyses were conducted to quantify accumulation of SA and its metabolites. Surprisingly, our results indicate that pathogen titer in the host did not change among widely disparate inoculation access periods (IAP) (7 day versus 1 continuous year). However, repeated monthly pulses of 7 day IAPs resulted in unexpectedly low pathogen titers in the host coincident with measurable plant defense responses induced by insect herbivory. Based on our results, we describe the dynamics of immune responses in *Citrus* following various intervals of vector feeding and frequencies of pathogen inoculation in the HLB pathosystem.

## Methods

### Controlled microcosm experiments

#### Plant husbandry

All experiments were performed using 2 year old *Citrus sinensis* L. Osbeck cv Valencia grafted onto US-812 rootstocks [35]. Plants were obtained from Southern Citrus Nurseries, Dundee, Florida. The process of eliminating possible insecticide residues from plants prior to initiation of experiments and in depth growing conditions have been described previously [23]. In brief, plants were maintained at 23 ± 3 °C, 60RH, and a 16:8 h (Light: Dark) photoperiod. Plants were watered twice per week and fertilized once per month with an alternating schedule of a 24–8-16 (Nitrogen–Phosphorus–Potassium) Miracle-Gro All Purpose Plant Food (Scotts Miracle-Gro Products, Marysville, OH) and a 10–10-10 (N–P–K) granular fertilizer (Growers Fertilizer Corp., Lake Alfred, FL).

#### Insect rearing conditions

Colonies of *Ca*Las-free and *Ca*Las-infected *D. citri* were maintained on *C. sinensis* L. Osbeck cv Valencia trees in two separate greenhouses. Each greenhouse was maintained at 26 ± 2 °C, 60–65% RH, and a 16:8 h (Light: Dark) photoperiod with a maximum photosynthetic radiation of 215 μmol s^− 1^ m^− 2^. The *Ca*Las infection rate was determined in each population monthly by testing a sub-sample of 40 adult insects per colony (infected and uninfected) using a TaqMan qPCR assay following the protocol described by Li et al. [36].

#### Inoculation frequency and herbivory duration treatments

Treatments were imposed on sweet orange, *C. sinensis*, seedling trees to simulate possible interactive scenarios of pathogen infection and insect herbivory that occur in citrus groves with HLB. For each scenario below, 6 replicate trees were challenged with *Ca*Las-infected or sham uninfected (control) psyllids. All trees (treatments and sham controls) were maintained individually in insect-proof cages (58.4 × 58.4 × 88.9 cm). Trees of similar phenology, characterized by presence of bud breaks and/or feather flush-like structures, were exposed to various frequencies of *Ca*Las inoculation access periods (IAP) and duration of insect herbivory, as follows:

i) First scenario: ‘one-time inoculation’ (OI): Plants (*N* = 6 plants) were exposed individually to groups of 20 *Ca*Las-infected (treatment) or *Ca*Las-free (control) *D. citri* adults (female: males; 1:1) for a 7 day IAP. On day 7, all eggs laid on flush shoots and *D. citri* adults were gently removed and *Ca*Las infection of adult psyllids was determined by TaqMan qPCR assay. This treatment simulated a scenario where a citrus grove is infested by a unique immigrant *D. citri* population that is eliminated by insecticide within 1 week under a zero-tolerance protocol.
ii) Second scenario: ‘pulsed inoculations’ (PI): Individual plants (*N* = 6 plants) were exposed monthly to 20 *Ca*Las-infected (treatment) or *Ca*Las-free (sham control) *D. citri* adults for a 7 day IAP. Psyllids were placed on branches within mesh sleeve cages. On the seventh day following insect release onto plants, all psyllids within cages were gently removed. This process was repeated monthly for the duration of the experiment (12 pulses of inoculations). In order to determine the *Ca*Las infection rate of the adult insects throughout the course of this experiment, a subset of 40 psyllids was collected and examined by TaqMan qPCR assay during each month of the experiment; the rate of *Ca*Las-infection in psyllids ranged between 35 and 72%. This treatment simulated a scenario where a citrus grove not harboring an endemic population of psyllids is periodically infested by immigrating psyllids, which are killed off by monthly applications of insecticides with short residual control.
iii) Third scenario: ‘continuous inoculations’ (CI): Individual plants (*N* = 6 plants) were exposed to an initial population of 20 *Ca*Las-infected (treatment) or *Ca*Las-free (sham control) *D. citri* by releasing psyllids into cages containing trees. In this treatment, insects were not removed, and plants were constantly exposed to continuously reproducing *D. citri* populations, ranging from 40 to 124 psyllids per plant. In order to estimate the *Ca*Las infection rate of the reproducing *D. citri* populations within cages, groups of insects (40/tree) were randomly collected at three specific time-points between August (2018) and February (2019), with rates of *Ca*Las infection of 25 and 55%, respectively. This treatment was designed to simulate a scenario where a grove is continuously infested by an endemic population *of D. citri* that is never eliminated by management practices.

Plant tissues were sampled monthly from the same 12 mature leaves on each replicate tree. Samples were collected by harvesting discs from these leaves beginning at the leaf tip and progressing down the petiole on each sampled leaf as described in [37, 38]. Plant tissue samples were immediately flash frozen in liquid nitrogen, ground with a tissue lyser, and stored at − 80 °C for further analyses.

### Genomic DNA extraction and *Ca*Las detection by TaqMan qPCR assays

To analyze the *Ca*Las infection rate in insects and plants, genomic DNA from individual *D. citri* adults and 20 mg of plant tissues was extracted using the DNeasy blood and tissue or DNeasy plant kits (Qiagen Inc., Valencia, CA), respectively. Both DNA extraction protocols were completed according to manufacturer’s instructions. Quantity of genomic DNA was determined in a Nanodrop 2000 Spectrophotometer (Thermo fisher Scientific, Waltham, MA), and samples were stored at − 20 °C until TaqMan qPCR analyses were performed.

TaqMan assays were conducted using specific TaqMan probes previously designed by Li et al. [36] and targeting the 16S rDNA for *Ca*Las, *Wingless* for *D. citri*, and *Cytochrome oxidase I* for *C. sinensis*. TaqMan qPCR reactions and conditions were carried out as previously described [38]. TaqMan reactions were performed in duplicates and positive reactions were considered for either target sequence if the cycle quantification (Cq) value was ≤36. For *C*Las detection analysis, ‘undetermined values’ were automatically assigned with a Cq value equal to 45 cycles.

### RNA extraction and cDNA synthesis

Total RNA extractions were carried out using 30 mg of ground plant tissue and the RNeasy Plant mini kit (Qiagen) according to the manufacturer’s instructions. To eliminate traces of genomic DNA, 1 μg of total RNA per sample was treated with DNase I using the Turbo DNase kit (Ambion), following the manufacturer’s protocol. RNA quantity and purity were analyzed in a Nanodrop 2000 Spectrophotometer (Thermo fisher Scientific, Waltham, MA).

cDNA synthesis was executed using the Verso cDNA Synthesis kit (Thermo Fisher scientific, CA). Each reaction consisted of: 500 ng of total RNA, 5X cDNA synthesis buffer, anchored-Oligo (dT) primers, RT Enhancer, and Verso Enzyme Mix following the manufacturer’s instructions. After synthesis, samples were stored at − 20 °C until further analyses.

### Selection of genes involved in SA signaling and metabolism

In order to examine the transcriptional regulation of seven genes involved in the SA-dependent defense responses on *C. sinensis*, we selected and evaluated four genes involved in the SA signaling; *Nonexpresser pathogenesis-related 1* (*NPR1*, accession number XM_006475416)*, Nonexpresser pathogenesis-related 3* (*NPR3*, accession number XM_006468378)*, Nonexpresser pathogenesis-related 4* (*NPR4*, accession number XM_025100491)*,* and the *Pathogenesis-related 1* (*PR1,* accession number XM_006486757). Also, we examined three genes related to SA metabolism: *Methylesterase 1* (*MES1,* accession number NM_127926)*, 2-oxoglutarate (2OG) and Fe(II)-dependent oxygenase* (*DMR6,* Accession number NM_117118) annotated and described in [39] as a *salicylic acid 3-hydroxylase,* and *S-adenosyl-L-methionine-dependent methyltransferases* (*BSMT1*, Accession number NM_111981). The oligonucleotide primers and expression patterns of these genes in response to feeding by uninfected *D. citri* were previously described [23].

### RT-qPCR reactions, gene expression, and metabolite analyses

RT-qPCR reactions were performed using an Applied Biosystems 7500 Real-Time PCR System (Thermo Fisher Scientific). Each reaction consisted of: 10 ng of cDNA (template), 300 nM of each gene-specific primer [23] and 1x of PowerUp™ SYBR® Green Master Mix; the final volume was adjusted with nuclease-free water to 20 μL. The real-time PCR program, conditions, normalization, and mathematical estimations of relative expression at different time-points were conducted according to Ibanez 2019 [23].

Accumulated SA and SA analytes were extracted and analyzed following the protocol and conditions described in [23]. In brief, SA metabolites were extracted using 20 mg of ground leaves and each sample was mixed with 0.25 mL of ice-cold methanol/water solution (20/80, v/v), including three internal standards (0.01 μg/mL salicylic acid-d_6_, for salicylic acid; 0.4 μg/mL 2,5-dihydroxybenzoic acid-d_3_, and 2,3-dihydroxybenzoic acid; and 0.8 μg/mL methyl salicylate-d_4_ for methyl salicylate). Samples were processed by ultra-sonic assisted extraction for 30 min in an ice block-filled bathtub. After incubation, samples were centrifuged at 30,000 g at 4 °C for 10 min, the supernatant was recovered and filtered using a 0.22 μm membrane filter, and 10 μL was injected into LC–MS/MS system. LC–MS/MS analyses were carried out with an Ultimate 3000 LC system coupled to a TSQ Quantiva triple quadrupole mass spectrometer (Thermo Fisher Scientific, San Jose, CA, USA). The analytes (salicylic acid 2-O-β-D-glucoside, 2,3-dihydroxybenzoic acid, salicylic acid, and methyl salicylate) were chromatographed on a Thermo Fisher scientific Acclaim C30 column (150 mm × 2.1 mm, 3.0 μm particle size) at a column temperature of 30 °C using a gradient elution with 0.1% formic acid in water (eluent A) and 0.1% formic acid in acetonitrile (eluent B). The gradient was as follows: 0–10 min in 20–95% solution of eluent A and 10–15 min in a 95% solution of eluent B. The column was re-equilibrated using the initial mobile phase before each subsequent run. The flow rate was set at 0.2 mL/min. The mass spectrometer was operated in both positive and negative electrospray ionization (ESI+ and ESI–) with selected reaction monitoring (SRM) mode. The analytes were assigned by comparing SRM transitions and retention times with authentic standards. Xcalibur software (Ver. 3.0) was employed for data processing and instrument control.

### Statistical analyses

To compare the amount of *Ca*Las DNA in mature leaves between inoculation treatments, and to determine the differences in gene expression and accumulation of SA and its metabolites, repeated measures analysis of variance (ANOVA) with Tukey’s test post hoc analyses were performed. All statistical analyses were run in RStudio environment [40].

## Results

### *C*Las detection in mature leaves of *C. sinensis*

The pattern of *Ca*Las-DNA titer in trees that were exposed to *Ca*Las-infected *D. citri* for a one-time, 7 day IAP was similar to that found in trees exposed to a reproducing population of *Ca*Las-infected *D. citri* (presence of eggs, nymphs, and adults) (Table 1). However, trees exposed to monthly pulses of 7 day IAPs using *Ca*Las-infected *D. citri* exhibited significantly lower *Ca*Las titer than the other two treatments for the duration of the experiment (12 months), with detectable levels of *Ca*Las DNA (0.010, 0.011 and 0.080 pg) at 240-, 330-, and 360 d, respectively. Based on these results, we chose time points when bacteria were detected in leaves (120-, 270-, and 360 d after first *Ca*Las-IAP) to analyze gene expression involved in SA-dependent defense response as well as the accumulation of SA and SA-metabolites.

**Table 1:** Quantification of *Ca*Las genomic DNA in mature leaves of *C. sinensis* after initiation of *Ca*Las-IAP. The time-points chosen for gene expression and metabolite analyses were highlighted in bold, italicized, and underlined. Data represent mean *Ca*Las titer ± standard deviation (SD) of six biological replicates per treatment. Different letters indicate statistical differences at *P* < 0.05

ρg of ***Ca***Las genomic DNA per 10 g of ***C. sinensis*** DNA			
Days after first ***Ca***Las-IAP	One-time inoculation	Pulsed inoculations	Continuous inoculations
30	N.D.	N.D.	N.D.
60	N.D.	N.D.	N.D.
90	N.D.	N.D.	N.D.
***120***	**0.715 ± 0.172**^**a**^	**N.D.**	**0.308 ± 0.130**^**a**^
150	N.D.	N.D.	N.D.
180	N.D.	N.D.	N.D.
210	N.D.	N.D.	N.D.
240	N.D.	0.010 ± 0.009^b^	N.D.
***270***	**0.680 ± 0.101**^**a**^	**N.D.**	**0.721 ± 0.270**^**a**^
300	N.D.	N.D.	0.041 ± 0.021^a^
330	0.662 ± 0.271^a^	0.011 ± 0.010^b^	0.818 ± 0.081^a^
***360***	**2.325 ± 1.193**^**d**^	**0.080 ± 0.005**^**c**^	**2.047 ± 0.602**^**d**^

*N.D* denotes No Detection of *Ca*Las DNA

### Gene expression and SA metabolite accumulation analyses

#### First scenario ‘one-time inoculation’

**Fig. 1:**
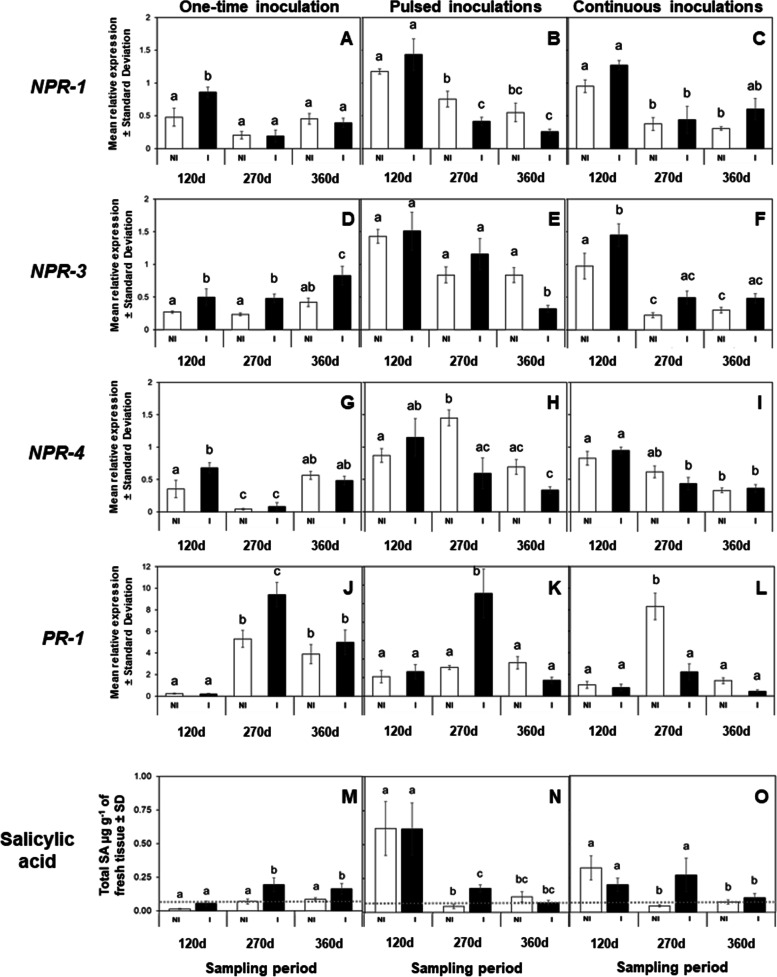
Varying frequencies of herbivory and *Ca*Las inoculation modulate SA-related gene expression and SA accumulation in mature leaves of *C. sinensis*. *Ca*Las infection and feeding by *D. citri* induces changes in expression patterns of *NPR1*, *NPR3*, *NPR4*, and *PR-1*, as well as, SA accumulation in mature leaves of *C. sinensis*. Expression patterns of genes involved in SA immune defense (**A-L**), SA accumulation in mature leaves (M-O). Expression levels were normalized using two references genes, *β-Actin* and *Elongation factor 1α*. Data represent mean relative expression ± standard deviation (SD) of six biological replicates per treatment. The gray dotted line denotes the level of SA accumulation at time zero in uninfected citrus plants. White bars represent plants exposed to Non-infected (NI) *D. citri*, while black bars denote plants exposed to *Ca*Las-infected (**I**) *D. citri*. Statistical analysis was performed with repeated measures ANOVA and Tukey post-hoc tests. Different letters indicate significant differences between the groups (*P* < 0.05)

Following the 7 day IAP treatment, the transcriptional regulation of *NPR1*, *NPR3*, *NPR4*, and *PR-1* and its association with endogenous SA levels were analyzed in leaves of *Ca*Las-infected and sham control plants (Fig. 1). Quantitative RT-PCR (qRT-PCR) analysis indicated that the relative expression of *NPR1* was only significantly upregulated in *Ca*Las-infected leaves during the first instance of *Ca*Las detection (120 d, *F = 8.4, df = 5, P* = 0.035) compared to the sham control (Fig. 1A). The relative expression of *NPR3* was significantly upregulated (120 d, *F = 7.72, df = 5, P* = 0.049; 270 d, *P* = 0.045; and 360 d, *P* = 0.007) during each of the three instances when *Ca*Las was detected in infected leaves as compared to non-infected samples (Fig. 1D). However, the relative expression of *NPR4* was significantly upregulated (*F = 12.15, df = 5, P* = 0.042) in *Ca*Las-infected leaves only during the first instance of detection (120 d), and its temporal expression pattern was downregulated (*P* < 0.05) during the second instance of *Ca*Las detection (270 d) compared to other time-points (Fig. 1G). The NPR-regulated gene, *PR-1*, was significantly upregulated during the second instance of *Ca*Las detection (270 d, *F = 20.67, df = 5, P* = 0.004) in *Ca*Las-infected leaves compared to sham control leaves (Fig. 1J), and its temporal expression was significantly upregulated (*P* < 0.05) during the second (270 d) and fourth (360 d) instances of *Ca*Las detection compared to the initial time when *Ca*Las was detected in this treatment.

The phytohormone SA binds to NPR proteins, modulating the transcription of the *PR-1* defense gene; significantly higher levels of SA were accumulated in *Ca*Las-infected leaves than in control leaves during the second (270 d, *F = 10.4, df = 5, P* = 0.006) and fourth (360 d, *P* = 0.049) instances of detection compared to the initial time (120 d) when *Ca*Las was detected in this treatment (Fig. 1M).

**Fig. 2:**
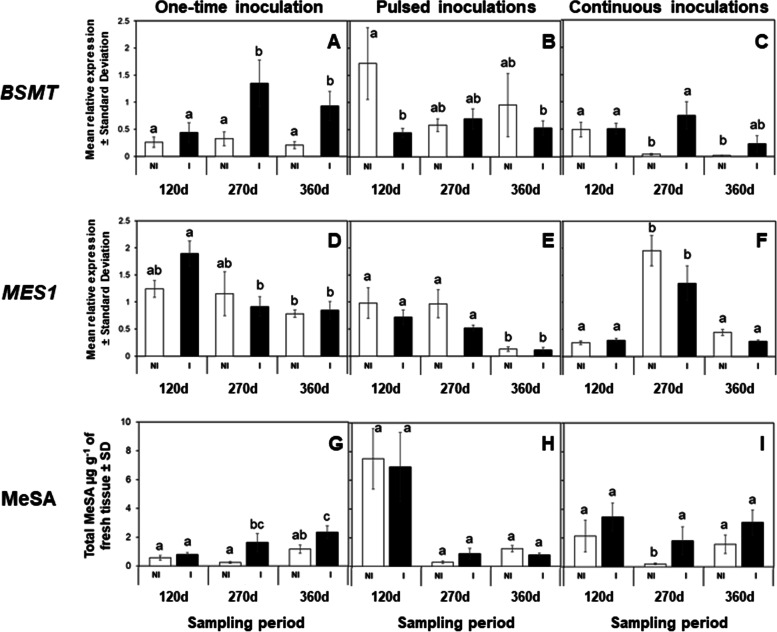
Varying frequencies of herbivory and *Ca*Las inoculation modulate genes involved in MeSA synthesis and metabolism and its accumulation in mature leaves of *C. sinensis*. *Ca*Las infection and feeding by *D. citri* induces changes in expression patterns of *BSMT* and *MES1* (**A-F**), as well as, MeSA accumulation in mature leaves of *C. sinensis* (**G-I**). Expression levels were normalized using two references genes, *β-Actin* and *Elongation factor 1α*. Data represent mean relative expression ± standard deviation (SD) of six biological replicates per treatment. White bars represent plants exposed to Non-infected (NI) *D. citri*, while black bars denote plants exposed to *Ca*Las-infected (**I**) *D. citri*. Statistical analysis was performed with repeated measures ANOVA and Tukey post-hoc tests. Different letters indicate significant differences between the groups (*P* < 0.05)

**Fig. 3:**
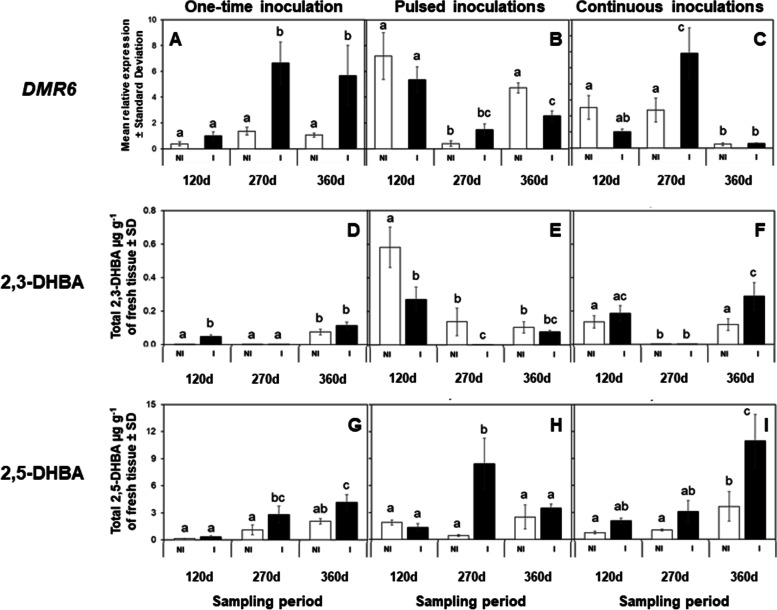
Varying frequencies of herbivory and *Ca*Las inoculation modulate the expression of *DMR6* and the accumulation SA hydroxylated molecules in mature leaves of *C. sinensis*. *Ca*Las infection and feeding by *D. citri* induces changes in expression patterns of *DMR6* (**A-C**), as well as, 2,3- and 2,5-DHBA accumulation in mature leaves of *C. sinensis* (**D-I**). Expression levels were normalized using two references genes, *β-Actin* and *Elongation factor 1α*. Data represent mean relative expression ± standard deviation (SD) of six biological replicates per treatment. White bars represent plants exposed to Non-infected (NI) *D. citri*, while black bars denote plants exposed to *Ca*Las-infected (**I**) *D. citri*. Statistical analysis was performed with repeated measures ANOVA and Tukey post-hoc tests. Different letters indicate significant differences between the groups (*P* < 0.05)

qRT-PCR analysis indicated that *BSMT,* required for the methylation of SA into MeSA, was significantly upregulated in *Ca*Las-infected mature leaves during the second (270 d, *F = 5.98, df = 5, P* = 0.039) and fourth (360 d, *P* = 0.049) instances of *Ca*Las detection compared to the sham control (Fig. 2A). However, *MES1*, requiring esterification of MeSA into SA (active defense hormone), did not exhibit statistical changes (*F = 2.50, df = 5*) in gene expression between *Ca*Las-infected and *Ca*Las-free leaves (Fig. 2d). *DMR6,* involved in hydroxylation of SA into 2,3- and 2,5-DHBA, was significantly upregulated in *Ca*Las-infected leaves during the second (270 d, *F = 8.49, df = 5, P* = 0.006) and fourth (360 d, *P* = 0.008) instances of *C*Las detection compared to *Ca*Las-free leaves (Fig. 3A).

MeSA was highly accumulated in *Ca*Las-infected leaves at the second (270 d, *F = 7.62, df = 5, P* = 0.015) and fourth (360 d, *P* = 0.032) instances of *Ca*Las detection. The hydroxylated molecules SA, 2,3- DHBA, and 2,5-DHBA exhibited a different temporal accumulation; the level of 2,3-DHBA was only significantly (*F = 23.48, df = 5, P* = 0.02) higher in *Ca*Las-infected than control leaves during the first (120 d) instance of *Ca*Las detection (Fig. 3D). However, accumulation of 2,5-DHBA was significantly higher in *Ca*Las-infected than in uninfected leaves during the second (*F = 10.21, df = 5,* 270 d, *P* = 0.049) and fourth (360 d, *P* = 0.047) instances of *Ca*Las detection in samples (Fig. 3G).

#### Second scenario: ‘pulsed inoculations’

qRT-PCR analysis of leaves from *C. sinensis* trees exposed to repeated (monthly) pulses of 7 day IAPs revealed that relative expression of *NPR1* was significantly downregulated in *Ca*Las-infected leaves during the second (270 d, *F = 13.04, df = 5, P* = 0.043) and fourth (360 d, *P* = 0.05) instances of *Ca*Las detection compared with their respective controls. Also, there was a significant reduction in the expression of *NPR1* over time in both uninfected and infected mature leaves (*P* < 0.05, Fig. 1B). Expression of *NPR3* was significantly downregulated in *Ca*Las-infected leaves at the fourth (360 d, *F = 7.58, df = 5, P* = 0.045) instance of *Ca*Las detection compared to the respective control (Fig. 1E). However, expression of *NPR4* was upregulated in uninfected leaves during the second (270 d, *F = 5.34, df = 5, P* = 0.032) instance of *Ca*Las detection relative to *Ca*Las-infected leaves (Fig. 1H). Overall, expression of all *NPR* genes tended to decrease over time in mature leaves as a response to pulses of 7 day IAPs by *Ca*Las-infected psyllids. Expression of *PR-1* was only upregulated in *Ca*Las-infected mature leaves during the second (270 d, *F = 6.44, df = 5, P* = 0.003) instance of *Ca*Las detection compared to its control at the same sampling interval (Fig. 1K).

Measurements of SA dynamics over time in trees that received pulses of 7-day IAPs revealed that SA was only significantly accumulated in *Ca*Las-infected mature leaves during the second (270 d, *F = 8.04, df = 5, P* = 0.049) instance of *Ca*Las detection compared to uninfected leaves. When temporal accumulation of SA was analyzed, we found that SA increased during the first (120 d) instance of *Ca*Las detection in both uninfected and infected leaves. Thereafter, there was a significant decline of SA in leaves observed during the second (270 d, *P* < 0.05) and fourth (360 d, *P* < 0.05) instances of *Ca*Las detection (Fig. 1N).

The expression of *BSMT* was downregulated (*F = 2.68, df = 5, P* = 0.049) during the first (120 d) instance of *Ca*Las detection in *Ca*Las-infected leaves as compared to that observed in the respective control (Fig. 2B). *BSMT* did exhibit significant changes in expression among sampling points over time (Fig. 2B). Expression of *MES1* did not differ between *Ca*Las-infected and uninfected leaves (Fig. 2E) at any of the sampling points; however, its expression decreased over time and was significantly (*P* < 0.05) lower in *Ca*Las-infected than uninfected leaves during the fourth (360 d) instance of *Ca*Las detection. Conversely, *DMR6* was significantly downregulated (*F = 9.43, df = 5, P* = 0.045) in *Ca*Las-infected leaves during the fourth (360 d) instance of *Ca*Las detection compared to that observed in uninfected leaves (Fig. 3B).

In addition to transcriptional analysis, we examined the temporal accumulation of SA metabolites. MeSA accumulation did not differ significantly (*F = 4.22, df = 5, P* > 0.05) between *Ca*Las-infected and uninfected leaves during the specific time points when *Ca*Las was detected. However, there were significant (*P* < 0.05) changes in MeSA over time with a greater accumulation during the first (120 d) instance of *Ca*Las detection compared to the other two time points when the pathogen DNA was detected (Fig. 2H). The analyte, 2,3-DHBA, was significantly lower in *Ca*Las-infected than control leaves during the first (120 d, *F = 5.52, df = 5, P* = 0.045) and second (270 d, *P* = 0.041) instances of *Ca*Las detection (Fig. 3E). Accumulation of 2,5-DHBA was significantly higher in *Ca*Las-infected than uninfected leaves during the second (270 d, *F = 6.81, df = 5, P* = 0.0001) instance of *Ca*Las detection (Fig. 3H).

#### Third scenario: ‘continuous inoculations’

The transcriptional regulation of *C. sinensis* exposed to a continuously reproducing population of *D. citri* revealed that *NPR1* expression did not differ between *Ca*Las-infected and uninfected leaves at any of the time points when *Ca*Las was detected (Fig. 1C). However, expression of *NPR1* was significantly (*F = 9.31, df = 5, P* < 0.05) lower during the second (270 d) and fourth (360 d) instances of *Ca*Las detection as compared to its expression when *Ca*Las was detected initially (120 d, Fig. 1C). The expression of *NPR3* was significantly upregulated in *Ca*Las-infected leaves during the first (120 d, *F = 15.37, df = 5, P* = 0.046) instance of *Ca*Las detection compared with that in its respective uninfected control (Fig. 1F), and similarly to *NPR1*, its expression was downregulated during the second (270 d) and fourth (360 d) instances of *Ca*Las detection in *Ca*Las-infected, as compared with uninfected, leaves (Fig. 1F). Expression of *NPR4* was significantly (*F = 11.31, df = 5, P* < 0.05) lower during the fourth (360 d) instance of *Ca*Las detection as compared its expression when *Ca*Las was detected initially (120 d, Fig. 1I). Expression of *PR-1* was significantly upregulated (*F = 22.58, df = 5, P* < 0.001) in uninfected than *Ca*Las-infected leaves during the second (270d) instance of *Ca*Las detection (Fig. 1L).

Accumulation of SA was significantly higher (*F = 4.42, df = 5*) during the second (270 d) instance of *Ca*Las detection in *Ca*Las-infected plants exposed to continuous re-inoculation and herbivory compared to that in complementary control plants that were exposed to uninfected insects. However, the concentration of SA was significantly (*P* > 0.05) lower during the fourth (360 d) instance of *Ca*Las detection, in both infected and uninfected leaves, than when *Ca*Las was detected initially (120 d, Fig. 1O).

Expression of *BMST* was significantly higher in *Ca*Las-infected than uninfected leaves during the second (270 d, *F = 5.23, df = 5*) instance of *Ca*Las detection in plants that were continuously exposed to reproducing *D. citri* (*P* = 0.002, Fig. 2C). The expression pattern of *MES1* did not differ between *Ca*Las-infected and uninfected samples; however, its expression was significantly upregulated (*F = 16.48, df = 5, P* < 0.05) during the second (270 d) instance of *Ca*Las detection in both treatments as compared to that observed during the other sampling points (Fig. 2F). The expression of the transcript involved in SA hydroxylation, *DMR6*, fluctuated over time with significantly (*F = 9.22, df = 5, P* = 0.007) higher expression in *Ca*Las-infected than uninfected leaves during the second (270 d) instance of *Ca*Las detection and lower overall expression during the fourth (360 d) instance of *Ca*Las detection in both treatments (*P* < 0.05, Fig. 3C).

The concentration of MeSA was significantly higher in *Ca*Las infected than uninfected leaves when both treatments were exposed to continuous herbivory only during the second (270 d, *F = 3.16, df = 5, P* < 0.05) instance of *Ca*Las detection (Fig. 2I). Of the hydroxylated SA metabolites quantified, 2,3-DHBA was significantly higher (*F = 9.67, df = 5, P* = 0.024) in leaves of plants exposed to *Ca*Las-infected insects than in plants exposed to uninfected insects during the fourth (360 d) instance of *Ca*Las detection (Fig. 3F). Also, 2,5-DHBA was only significantly higher in *Ca*Las-infected than uninfected leaves during the fourth (360 d) instance of *Ca*Las detection (*F = 13.35, df = 5, P* = 0.001), and there was a trend of progressively increasing 2,5-DHBA in leaves exposed to both infected and uninfected insects over time (Fig. 3I).

### Comparative analyses of SA and its analytes in response to the interaction of insect herbivory and pathogen infection

**Fig. 4:**
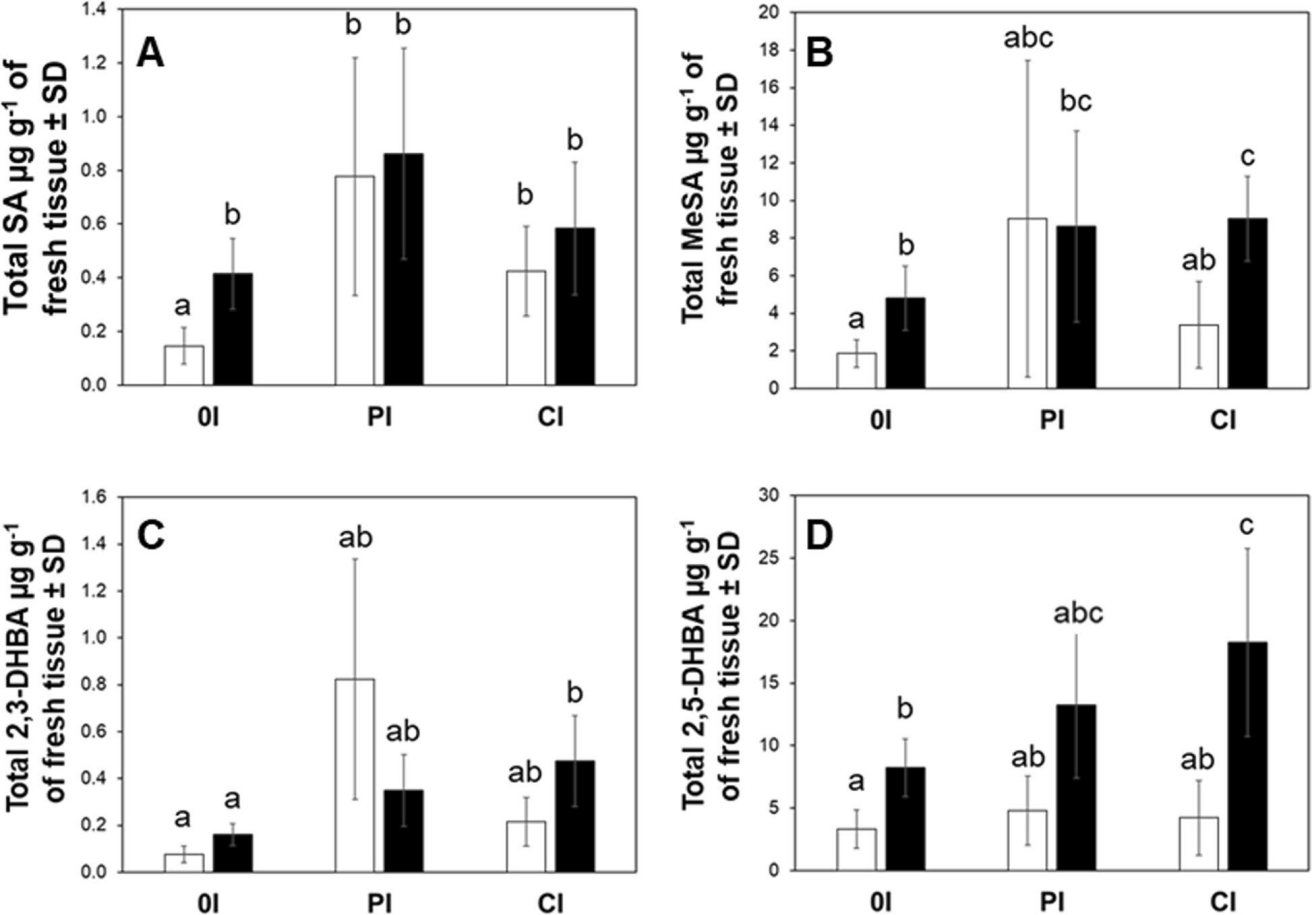
Cumulative concentration of SA and its metabolites among different scenarios of a vector-host-pathogen interaction in citrus. Data represent the cumulative mean concentration of each molecule ± standard deviation (SD) of six biological replicates per treatment. Statistical analysis was performed with ANOVA with Tukey post-hoc tests. Different letters indicate significant differences between the samples (*P* < 0.05). White bars represent citrus plants exposed to uninfected psyllids, and black bars denote plants challenged with *Ca*Las-infected psyllids. Abbreviations: OI = One-time inoculation, PI = Pulsed inoculation, and CI = continuous inoculation

When accumulation of SA and its metabolites (MeSA, 2,3- and 2,5- DHBA) was compared among the three scenarios investigated, there was a statistically significant (*F = 5.38, df = 5, P = 0.002*) increase of SA in *Ca*Las-infected leaves of plants exposed to infected *D. citri* than that observed with the uninfected, sham treatment in the one-time inoculation scenario (Fig. 4), suggesting a *Ca*Las-induced SA response. However, there was greater overall accumulation of SA in mature leaves from trees under the pulsed and continuous inoculation scenarios than that accumulating in the one-time inoculation scenario. Furthermore, when comparing between pulsed and continuous inoculation scenarios, a difference in accumulation of SA between the *Ca*Las infection treatment and sham control was not apparent (Fig. 4A). However, accumulation of MeSA was significantly higher (OI, *F = 2.70, df = 5, P = 0.003, and CI, P = 0.01*) in the one-time and continuous inoculation scenarios in *Ca*Las-infected than in uninfected, sham-treated plants. When the accumulation of SA hydroxylated metabolites were analyzed, accumulation of 2,3-DHBA did not differ (*F = 7.82, df = 5*, *P > 0.05*) between *Ca*Las-infected and uninfected, sham-treated plants (OI, PI, and CI). In contrast, significantly higher (*F = 10.22, df = 5*), accumulation of 2,5-DHBA and 2,5-DHBA was observed in the one-time and continuous inoculation scenarios in *Ca*Las-infected than sham-treated, control plants (*P = 0.01*, and *P = 0.02*, respectively; Fig. 4D).

## Discussion

Plants perceive microbial and insect molecules as danger signals and mount defense against invasions. Studies investigating plant interactions with phytopathogens have established that plants have a layered innate immune system which responds to different microbial elicitors [5], and early signaling events are similar to those induced by insects [41–44]. The current investigation attempted to both disentangle the contributing effects of insect herbivory and pathogen infection on host plant response, as well as gain insights into their interactive effects on disease progression. We challenged citrus hosts with insect and/or pathogen activity by manipulative treatments designed to mimic the types of infestations/infections that citrus growers experience when cultivating citrus in the face of Huanglongbing (HLB). *C. sinensis* plants were challenged with three putative scenarios of vector-host-pathogen interactions [one time (OI), pulsed inoculation (PI), and continuous inoculation (CI)] to explore transcriptional and metabolic modulation of SA-dependent defense responses in the host after varying frequencies of herbivore infestation and/or *Ca*Las inoculation. Moreover, the investigation was longitudinal, which allowed sampling of gene expression and metabolite accumulation over the course of disease progression.

Progression of pathogen in the host after trees were exposed to various frequencies of vector IAP (with or without pathogen present) was measured by monitoring *Ca*Las DNA. Simultaneously, we measured defense responses in *C. sinensis*, which may combat pathogenesis induced by *Ca*Las. Surprisingly, our results indicate that *C. sinensis* trees challenged by *Ca*Las-infected insects for only a 7 day IAP exhibited similar temporal dynamics of *Ca*Las titer to trees that were continuously challenged with infected *D. citri* for over 1 yr. In contrast, trees exposed to repeated (pulsed-inoculations, 12 times) 7 day IAP challenges by infected *D. citri* exhibited the lowest *Ca*Las titer, suggesting putative short-term plant defense responses to *Ca*Las (HLB progression, infection). We postulate that SA-dependent defense responses were active in these trees to reduce or ‘control’ *Ca*Las colonization, but this hypothesis requires further investigation.

Our results suggest that herbivory played an important role in inducing synthesis and accumulation of SA in mature leaves of *C. sinensis*, whether or not those trees are coincidentally infected with *Ca*Las. Accumulation of SA and the SA-signaling pathway both play a critical role in plant defenses against pathogens [20] and in response to phloem-feeding herbivores [21–23]. SA regulates PTI and ETI defense mechanisms which leads to activation of systemic acquired resistance (SAR). We postulate that it might be eventually possible to manipulate natural defense and immune responses of *Citrus* to *Ca*Las and/or *D. citri* with sufficient understanding of the genes involved in SA-dependent immune response (*NPR1*, *NPR3*, *NPR4*, and *PR-1*) and modulation of downstream metabolic modifications (*BMST*, *MES1*, and *DMR6*).

Previously, a gene involved in the hydroxylation of SA, *SA hydroxylase*, was identified and characterized in the *Ca*Las genome; infected plants exhibited significantly lower SA accumulation in leaves than uninfected plants 8 months after graft inoculation [45]. However, a similar reduction in SA was not observed in other investigations of the *Ca*Las-*C. sinensis* interaction. For example, Lu et al. [46] showed that symptomatic and PCR-positive leaves collected 3–4 months after graft-inoculation exhibited higher SA accumulation than leaves from uninfected trees. Similar results were detected after ~ 6.4 months in young and mature leaves from trees previously graft-inoculated with *Ca*Las [47]. In trees that were not infested with insects, but were inoculated with *Ca*Las (one-time inoculation), we found significantly greater accumulation of SA in leaves of infected than uninfected trees at 270- and 360 days after experiments were initiated (Fig. 1M). This outcome suggests that *Ca*Las did not evade SA-mediated immune responses via activity of its encoded SA hydroxylase within the context of this scenario where pathogen occurred *in planta* in the absence of concurrent insect herbivory/re-inoculation.

In addition to upstream regulation of SA, this hormone is also modulated by downstream metabolic modifications, including glycosylation, methylation, amino acid conjugation, and hydroxylation [25, 27]. In this investigation, production of the methylated (MeSA) and hydroxylated SA-metabolites (2,3- and 2,5-DHBAs) was measured longitudinally (Figs. 1, 2 and 3) and their cumulative amounts were determined for the duration of the experiment (Fig. 4B-D). MeSA serves multiple functions, including triggering of SAR in response to microbial pathogens as shown in Arabidopsis, tobacco, and potato [48–51]. MeSA also functions as an indirect defense cue by attracting natural enemies in response to insect feeding [52–54]; e.g. MeSA attracts *D. citri* to citrus trees [55]. We measured substantial accumulation of MeSA in *Ca*Las-infected leaves (one-time and pulsed inoculation scenarios). When plants were challenged with repeated pulses of the *Ca*Las-infected vectors, the titer of *Ca*Las in infected plants was the lowest of all three infection scenarios investigated (Table 1). In trees subjected to this IAP scenario, the concentration of MeSA varied over time and no difference in concentration was observed between trees injured by *Ca*Las-infected and uninfected insects (Fig. 4B). In contrast, when plants were subjected to a one-time IAP (one-time inoculation scenario) or a continuously breeding population of vectors (continuous inoculations), the concentration of MeSA was substantially higher in leaves from trees that were also challenged with the pathogen than in the sham controls that received only insect injury in the absence of *Ca*Las. These results are congruent with previous observations showing that *Ca*Las-infected trees are attractive to *D. citri*, which is in part explained by MeSA serving an attractant cue to the vector [55].

Although the specific roles played by the hydroxylated SA molecules 2,3- and 2,5-DHBA in plant immune defense have not yet been clearly elucidated, studies suggest that exogenous applications of 2,3-DHBA in Arabidopsis induced weak *PR-1* expression [56]. Furthermore, 2,5-DHBA induced the synthesis of a different set of PR proteins in tomato [57]. We found no difference in 2,3-DHBA accumulation between trees that were subjected to sham (uninfected) versus *Ca*Las-infected IAPs, which resulted in eventual tree infection. However, more 2,3-DHBA accumulated in *Ca*Las-infected leaves that were subjected to a continuously breeding population of vectors (continuous inoculation scenario) than in *Ca*Las-infected leaves from trees that were only injured by vectors during an initial one-time 7 day IAP (one-time inoculation scenario, Fig. 4C). In the case of 2,5-DHBA, accumulation was higher in *Ca*Las-infected leaves (both one-time and continuous inoculation scenarios) than their respective sham controls, while the pulsed inoculation scenario caused intermediate accumulation with no difference observed between *Ca*Las-infected and uninfected (sham IAP) trees (Fig. 4D). Based on our results, it appears that *Ca*Las infection induces the accumulation of 2,5-DHBA in *C. sinensis*; however, the effect of this analyte within the context of plant immune response is still unclear. The elucidation of this function was beyond the scope of our current study, but the role(s) of these hydroxylated SA molecules in *Citrus* should be investigated further.

### Gene expression and its association with SA and related metabolites

We propose a working model of SA-modulated defense mechanisms in *C. sinensis* (Supplementary Fig. 1) that examines the expression patterns of *NPR1*, *NPR3*, *NPR4*, *PR-1*, *BMST*, *MES1*, and *DMR6*, as well as the accumulation of SA and its hydroxylated and methylated analytes based on the three scenarios of vector interactions with the host and pathogen (one-time, pulsed, and continuous inoculations).

#### One-time inoculation scenario

Using Arabidopsis and other plant species as models, it has been proposed that overexpression of *NPR1* induces expression of disease response genes, such as *PR-1* [58–60]. In our first scenario, *Ca*Las-infected samples exhibited a transient upregulation of *NPR1* in mature leaves only at 120 days after initial IAP and this coincided with the initial detection of *Ca*Las in the leaf tissue. However, upregulation of *NPR1* was not associated with a similar *PR-1* expression pattern as expected; *PR-1* was upregulated in *Ca*Las-infected leaves much later after initial IAP at 270- and 360- days, which was also much later than the initial detection of *Ca*Las titer in leaves of infected trees (day 120). Furthermore, this *PR-1* expression pattern was associated with high accumulation of SA in *Ca*Las-infected samples and reduced expression of *NPR4*. Recently, it was shown in Arabidopsis that the transcription factors NPR3/NPR4 interact with TGA2/TGA5/TGA6 to downregulate the expression of genes involved in plant defenses when plants are not infected and exhibit low SA concentration. Also, a gain-of-function mutation, *npr4-4D*, leads to a SA-insensitive mutant protein that represses SA-inducible defense genes [34]. Consequently, the expression pattern of *PR-1* was congruent with low expression of *NPR4* in association with SA accumulation in *Ca*Las-infected leaves. Therefore, we suggest that infection of *C. sinensis* with *Ca*Las activates SA-dependent defense responses even in the absence of phloem feeding injury caused by the vector. However, characterizing how the pathogen induces this SA response in *C. sinensis* would likely require investigations employing gain/loss of function mutations, which will only be possible once this fastidious bacterium (*Ca*Las) is reliably cultured [61–65].

#### Pulsed-inoculations scenario

For this inoculation scenario, trees were challenged by monthly ‘pulses’ of 7 day IAPs inflicted by *Ca*Las-infected adult psyllids. To our surprise, these trees appeared to exhibit a much higher degree of ‘tolerance’ against *Ca*Las colonization, given that the lowest pathogen titers were measured in these trees when comparing across all three infection scenarios that were examined. In the pulsed inoculation scenario, SA-dependent immune responses and associated accumulation of SA and its metabolites were both substantial at 120 days after initial IAP, but then progressively decreased over time (Figs. 2 and 3). We postulate that repeated exposure to insect injury caused sufficient accumulation of SA in *C. sinensis* to inhibit *Ca*Las colonization. Furthermore, we observed high expression of *PR-1* coincident with downregulation of *NPR1* and *NPR4* in trees exposed to *Ca*Las-infected insects by 270 days after initial IAP (Fig. 1) even though no bacterial DNA from the pathogen was detected in leaf phloem at this point in the experiment (Table 1).

#### Continuous inoculations scenario

In this scenario, trees were challenged by the possibility of continuous *Ca*Las re-infection and constant injury inflicted by a reproducing population of *D. citri*. In general, the pattern of *NPR* transcripts differed from the one-time inoculation scenario in that it decreased over time in both uninfected (sham inoculation receiving insect injury only) and *Ca*Las-infected trees; this downregulation of *NPR* response was also associated with lower SA accumulation than in the one-time inoculation scenario. Specifically, trees that were exposed to continuous feeding by *D. citri* initially accumulated SA (120 days after initial IAP), but SA production decreased significantly at later time-points (270- and 360- days after initial IAP). Injury to *C. sinensis* by *D. citri* feeding for brief intervals (≤14 days) and in the absence of *Ca*Las infection induces expression of *NPR1* and *PR-1*, but is not associated with changes in SA accumulation compared to control plants (without *D. citri*) [23]. In contrast, prolonged (≥ 150 days) feeding injury by uninfected *D. citri* stimulates accumulation of SA, yet no changes in expression of *NPR1* and *PR-1* were detected in these trees [23]. Collectively, these results indicate that psyllid feeding injury stimulates immune defenses in *C. sinensis* soon after feeding injury is initiated which can result in accumulation of SA, but prolonged feeding turns off SA synthesis and/or increases expression of genes involved in SA metabolism (*BMST* and *DMR6*), resulting in accumulation of SA metabolites (MeSA, 2,3-DHBA, and 2,5-DHBA) (Fig. 4).

Our investigation describes gene expression associated with SA-dependent immune responses, as well as resultant accumulation of SA and its metabolites in *C. sinensis* after various infection scenarios with the pathogen causing HLB. The investigation considered the interaction between the vector and/or pathogen on host responses and varied duration of exposure to these stressors (alone or in combinations). Based on our findings, we postulate that SA-dependent defense responses were activated in *Citrus* trees exposed to pulses of *Ca*Las inoculation at least until the initial instance of *Ca*Las detection in leaves. SA responses are of particular relevance to understanding HLB disease progression in sweet oranges, given that increased callose and phloem protein 2 deposition occurs in sieve elements of citrus trees expressing HLB symptoms [66, 67]. A recent investigation by Deng et al. [4], showed that ‘Bearss’ lemon and ‘LB8–9’ Sugar Belle® mandarin were more tolerant to *Ca*Las infection than most commercial citrus varieties, which was associated with lower levels of phloem disruption and greater phloem regeneration than in HLB-susceptible sweet orange. It is unclear whether SA was associated with this phenomenon; thus, it is imperative to understand the temporal accumulation of SA in citrus exposed to *D. citri* (vector) herbivory, as well as *Ca*Las (pathogen) infection, and use this information to address fundamental questions related to symptom suppression and associated practical questions related to development of tree therapies. Determining gene function involved in immune responses and SA modification in *Citrus* may yield novel genotypes that express tolerance against *Ca*Las and/or modify vector behavior to reduce inoculation.

The abnormal gene expression and levels of metabolite accumulation observed in trees challenged with prolonged herbivory indicates that *D. citri* injury causes deleterious effects on immune response in *C. sinensis*. From an immediately practical perspective, these findings support the practice of vector suppression as part of HLB management in citrus, even in areas where HLB is established endemically [23]. Among our ongoing and future investigations, characterization of phenotypic traits in plants associated with *Ca*Las infection and/or psyllid infestations under the scenarios investigated here remains a priority. As an example, such results may allow development of tree stress-based decision tools using image-based phenotyping and machine learning.

## Conclusions

We explored tritrophic interactions among an insect vector (*Diaphorina citri*) – phytopathogen (*Candidatus* Liberibacter asiaticus) – and cultivated fruit crop [sweet orange, *Citrus sinensis* (L) Osbeck]. Transcriptional and metabolic responses of plants were analyzed over an extended time-course of disease progression after various frequencies of herbivore feeding and durations of pathogen infection using manipulative treatments designed to mimic the types of infestations/infections that citrus growers experience when cultivating citrus in the presence of the devastating citrus disease, Huanglongbing. We determined that in the absence of coincident psyllid feeding damage, citrus trees could activate a defense response against the pathogen by inducing the salicylic acid (SA) pathway through downregulation of *NPR4* and upregulation of *PR-1* expression. Repeated, monthly ‘pulses’ of herbivory led to pronounced accumulation of SA that was coincident with almost undetectable pathogen titers in plants. Although insect injury initially activated SA-dependent defense responses, continuous and/or long-term (≥ 270 days) herbivory shut down *PR-1*-dependent defense responses against the pathogen. Our results offer a mechanism explaining how vector suppression contributes to maintaining health of cultivated citrus in areas where Huanglongbing is endemic. Our results also point to specific gene targets that may yield novel genotypes expressing tolerance against *Ca*Las after appropriate manipulations.

## Supplementary Information

**Additional file 1: S1 Fig.** Proposed models of SA-dependent immune responses in *C. sinensis*. The figure was created and designed by the authors.

## Data Availability

Data and materials are available on request from the corresponding author. This study does not use large datasets consequently no supplementary data has been deposited in public repositories.

## Abbreviations

SA: Salicylic acid
HLB: Huanglongbing
MeSA: SA methyl ester (methyl salicylate)
2,3-DHBA: 2,3-Dihydroxybenzoic acid
2,5-DHBA: 2,5-Dihydroxybenzoic acid
ACP: Asian citrus psyllid
BSMT: Benzoic acid/salicylic acid carboxyl methyltransferase
MES1: Methylesterase 1
NPR1: Nonexpresser of Pathogenesis Related genes 1
NPR3: Nonexpresser of Pathogenesis Related genes 3
NPR4: Nonexpresser of Pathogenesis Related genes 4
PR-1: Pathogenesis Related gene 1
SAR: Systemic acquired resistance
*Ca*Las: *Candidatus* Liberibacter asiaticus

## References

[1] Bové JM (2006). Huanglongbing: a destructive, newly emerging, century-old disease of citrus. J Plant Pathol.

[2] Manjunath K, Halbert S, Ramadugu C, Webb S, Lee R (2008). Detection of ‘*Candidatus* Liberibacter asiaticus’ in *Diaphorina citri* and its importance in the management of citrus huanglongbing in Florida. Phytopathology..

[3] Spreen TH, Baldwin J-P, Futch SH (2014). An economic assessment of the impact of Huanglongbing on citrus tree plantings in Florida. HortScience..

[4] Deng H, Achor DS, Etxeberria E, Yu Q, Du D, Stanton D (2019). Phloem regeneration is a mechanism for Huanglongbing-tolerance of ‘Bearss’ lemon and ‘LB8-9’ sugar belle® mandarin. Front Plant Sci.

[5] Jones JD, Dangl JL (2006). The plant immune system. Nature..

[6] Boller T, Felix G (2009). A renaissance of elicitors: perception of microbe-associated molecular patterns and danger signals by pattern-recognition receptors. Annu Rev Plant Biol.

[7] Dodds PN, Rathjen JP (2010). Plant immunity: towards an integrated view of plant–pathogen interactions. Nat Rev Genet.

[8] Nicaise V, Roux M, Zipfel C (2009). Recent advances in PAMP-triggered immunity against bacteria: pattern recognition receptors watch over and raise the alarm. Plant Physiol.

[9] Tsuda K, Katagiri F (2010). Comparing signaling mechanisms engaged in pattern-triggered and effector-triggered immunity. Curr Opin Plant Biol.

[10] Monaghan J, Zipfel C (2012). Plant pattern recognition receptor complexes at the plasma membrane. Curr Opin Plant Biol.

[11] Greenberg JT, Yao N (2004). The role and regulation of programmed cell death in plant–pathogen interactions. Cell Microbiol.

[12] McHale L, Tan X, Koehl P, Michelmore RW (2006). Plant NBS-LRR proteins: adaptable guards. Genome Biol.

[13] Dangl JL, Jones JD (2001). Plant pathogens and integrated defense responses to infection. Nature..

[14] Chinchilla D, Zipfel C, Robatzek S, Kemmerling B, Nürnberger T, Jones JD (2007). A flagellin-induced complex of the receptor FLS2 and BAK1 initiates plant defense. Nature..

[15] Heese A, Hann DR, Gimenez-Ibanez S, Jones AM, He K, Li J (2007). The receptor-like kinase SERK3/BAK1 is a central regulator of innate immunity in plants. Proc Natl Acad Sci U S A.

[16] Shan L, He P, Li J, Heese A, Peck SC, Nürnberger T (2008). Bacterial effectors target the common signaling partner BAK1 to disrupt multiple MAMP receptor-signaling complexes and impede plant immunity. Cell Host Microbe.

[17] Zhang J, Shao F, Li Y, Cui H, Chen L, Li H (2007). A *pseudomonas syringae* effector inactivates MAPKs to suppress PAMP-induced immunity in plants. Cell Host Microbe.

[18] Glazebrook J (2005). Contrasting mechanisms of defense against biotrophic and necrotrophic pathogens. Annu Rev Phytopathol.

[19] Mescher MC, De Moraes CM (2014). Role of plant sensory perception in plant–animal interactions. J Exp Bot.

[20] Walling LL (2008). Avoiding effective defenses: strategies employed by phloem-feeding insects. Plant Physiol.

[21] Casteel CL, Hansen AK, Walling LL, Paine TD (2012). Manipulation of plant defense responses by the tomato psyllid (*Bactericerca cockerelli*) and its associated endosymbiont *Candidatus* Liberibacter psyllaurous. PLoS One.

[22] Huot OB, Levy JG, Tamborindeguy C (2018). Global gene regulation in tomato plant (*Solanum lycopersicum*) responding to vector (*Bactericera cockerelli*) feeding and pathogen (‘*Candidatus* Liberibacter solanacearum’) infection. Plant Mol Biol.

[23] Ibanez F, Suh JH, Wang Y, Stelinski LL (2019). Long-term, sustained feeding by Asian citrus psyllid disrupts salicylic acid homeostasis in sweet orange. BMC Plant Biol.

[24] Chen Z, Zheng Z, Huang J, Lai Z, Fan B (2009). Biosynthesis of salicylic acid in plants. Plant Signal Behav.

[25] D'Maris Amick Dempsey AC, Vlot MCW, Daniel FK (2011). Salicylic acid biosynthesis and metabolism. Arabidopsis Book Am Soc Plant Biol.

[26] Rekhter D, Lüdke D, Ding Y, Feussner K, Zienkiewicz K, Lipka V (2019). Isochorismate-derived biosynthesis of the plant stress hormone salicylic acid PBS3 is the missing link in plant-specific isochorismate-derived salicylic acid biosynthesis. Science..

[27] Maruri-López I, Aviles-Baltazar NY, Buchala A, Serrano M (2019). Intra and extracellular journey of the phytohormone salicylic acid. Front Plant Sci.

[28] Huang X-x, Zhu G-q, Liu Q, Chen L, Li Y-j, Hou B-k (2018). Modulation of plant salicylic acid-associated immune responses via glycosylation of dihydroxybenzoic acids. Plant Physiol.

[29] Zhou J-M, Trifa Y, Silva H, Pontier D, Lam E, Shah J (2000). NPR1 differentially interacts with members of the TGA/OBF family of transcription factors that bind an element of the *PR-1* gene required for induction by salicylic acid. Mol Plant-Microbe Interact.

[30] Wang D, Amornsiripanitch N, Dong X (2006). A genomic approach to identify regulatory nodes in the transcriptional network of systemic acquired resistance in plants. PLoS Pathog.

[31] Delaney T, Friedrich L, Ryals J (1995). Arabidopsis signal transduction mutant defective in chemically and biologically induced disease resistance. Proc Natl Acad Sci U S A.

[32] Fu ZQ, Yan S, Saleh A, Wang W, Ruble J, Oka N (2012). NPR3 and NPR4 are receptors for the immune signal salicylic acid in plants. Nature..

[33] Yan S, Dong X (2014). Perception of the plant immune signal salicylic acid. Curr Opin Plant Biol.

[34] Ding Y, Sun T, Ao K, Peng Y, Zhang Y, Li X (2018). Opposite roles of salicylic acid receptors *NPR1* and *NPR3/NPR4* in transcriptional regulation of plant immunity. Cell..

[35] Bowman KD, Rouse RE (2006). US-812 citrus rootstock. HortScience..

[36] Li W, Hartung JS, Levy L (2006). Quantitative real-time PCR for detection and identification of *Candidatus* Liberibacter species associated with citrus huanglongbing. J Microbiol Methods.

[37] Etxeberria E, Gonzalez P, Vincent C, Schumann A (2017). An improved method to track changes of *Candidatus* Liberibacter asiaticus titer in HLB-affected Citrus trees: extended persistence of detectable CLas DNA after cell death. Proc Florida State Horticult Soc.

[38] Ibanez F, Stelinski LL (2019). Temporal dynamics of *Candidatus* Liberibacter asiaticus titer in mature leaves from *Citrus sinensis* cv Valencia are associated with vegetative growth. J Econ Entomol.

[39] Zhang K, Halitschke R, Yin C, Liu C-J, Gan S-S (2013). *Salicylic acid 3-hydroxylase* regulates Arabidopsis leaf longevity by mediating salicylic acid catabolism. Proc Natl Acad Sci U S A.

[40] RStudio (2015). RStudio: integrated development for R.

[41] Walling LL (2000). The myriad plant responses to herbivores. J Plant Growth Regul.

[42] Taylor JE, Hatcher PE, Paul ND (2004). Crosstalk between plant responses to pathogens and herbivores: a view from the outside in. J Exp Bot.

[43] Garcia-Brugger A, Lamotte O, Vandelle E, Bourque S, Lecourieux D, Poinssot B (2006). Early signaling events induced by elicitors of plant defenses. Mol Plant-Microbe Interact.

[44] Maffei ME, Mithöfer A, Boland W (2007). Before gene expression: early events in plant–insect interaction. Trends Plant Sci.

[45] Li J, Pang Z, Trivedi P, Zhou X, Ying X, Jia H (2017). ‘*Candidatus* Liberibacter asiaticus’ encodes a functional *salicylic acid (SA) hydroxylase* that degrades SA to suppress plant defenses. Mol Plant-Microbe Interact.

[46] Lu H, Zhang C, Albrecht U, Shimizu R, Bowman K (2013). Overexpression of a citrus *NDR1* ortholog increases disease resistance in Arabidopsis. Front Plant Sci.

[47] Nehela Y, Hijaz F, Elzaawely AA, El-Zahaby HM, Killiny N (2018). Citrus phytohormonal response to *Candidatus* Liberibacter asiaticus and its vector *Diaphorina citri*. Physiol Mol Plant Pathol.

[48] Park S-W, Kaimoyo E, Kumar D, Mosher S, Klessig DF (2007). Methyl salicylate is a critical mobile signal for plant systemic acquired resistance. Science..

[49] Vlot AC, Liu PP, Cameron RK, Park SW, Yang Y, Kumar D (2008). Identification of likely orthologs of tobacco *salicylic acid-binding protein 2* and their role in systemic acquired resistance in Arabidopsis thaliana. Plant J.

[50] Vlot AC, Dempsey DMA, Klessig DF (2009). Salicylic acid, a multifaceted hormone to combat disease. Annu Rev Phytopathol.

[51] Manosalva PM, Park S-W, Forouhar F, Tong L, Fry WE, Klessig DF (2010). Methyl esterase 1 (StMES1) is required for systemic acquired resistance in potato. Mol Plant-Microbe Interact.

[52] Van Poecke RM, Posthumus MA, Dicke M (2001). Herbivore-induced volatile production by *Arabidopsis thaliana* leads to attraction of the parasitoid *Cotesia rubecula*: chemical, behavioral, and gene-expression analysis. J Chem Ecol.

[53] Simpson M, Gurr GM, Simmons AT, Wratten SD, James DG, Leeson G (2011). Insect attraction to synthetic herbivore-induced plant volatile-treated field crops. Agric For Entomol.

[54] Martini X, Pelz-Stelinski KS, Stelinski LL (2014). Plant pathogen-induced volatiles attract parasitoids to increase parasitism of an insect vector. Front Ecol Evol.

[55] Mann RS, Ali JG, Hermann SL, Tiwari S, Pelz-Stelinski KS, Alborn HT (2012). Induced release of a plant-defense volatile ‘deceptively’ attracts insect vectors to plants infected with a bacterial pathogen. PLoS Pathog.

[56] Bartsch M, Bednarek P, Vivancos PD, Schneider B, von Roepenack-Lahaye E, Foyer CH (2010). Accumulation of isochorismate-derived 2, 3-dihydroxybenzoic 3-O-β-D-xyloside in Arabidopsis resistance to pathogens and aging of leaves. J Biol Chem.

[57] Bellés JM, Garro R, Fayos J, Navarro P, Primo J, Conejero V (1999). Gentisic acid as a pathogen-inducible signal, additional to salicylic acid for activation of plant defenses in tomato. Mol Plant-Microbe Interact.

[58] Cao H, Bowling SA, Gordon AS, Dong X (1994). Characterization of an Arabidopsis mutant that is nonresponsive to inducers of systemic acquired resistance. Plant Cell.

[59] Chern M, Fitzgerald HA, Canlas PE, Navarre DA, Ronald PC (2005). Overexpression of a rice *NPR1* homolog leads to constitutive activation of defense response and hypersensitivity to light. Mol Plant-Microbe Interact.

[60] Le Henanff G, Farine S, Kieffer-Mazet F, Miclot A-S, Heitz T, Mestre P (2011). *Vitis vinifera VvNPR1* is the functional ortholog of *AtNPR1* and its overexpression in grapevine triggers constitutive activation of *PR* genes and enhanced resistance to powdery mildew. Planta..

[61] Davis MJ, Mondal SN, Chen H, Rogers ME, Brlansky RH (2008). Co-cultivation of ‘*Candidatus* Liberibacter asiaticus’ with actinobacteria from citrus with huanglongbing. Plant Dis.

[62] Sechler A, Schuenzel E, Cooke P, Donnua S, Thaveechai N, Postnikova E (2009). Cultivation of ‘*Candidatus* Liberibacter asiaticus’,‘*Ca*. *L. africanus*’, and ‘*Ca*. *L. americanus*’ associated with huanglongbing. Phytopathology..

[63] Tyler HL, Roesch LF, Gowda S, Dawson WO, Triplett EW (2009). Confirmation of the sequence of ‘*Candidatus* Liberibacter asiaticus’ and assessment of microbial diversity in Huanglongbing-infected citrus phloem using a metagenomic approach. Mol Plant-Microbe Interact.

[64] Parker JK, Wisotsky SR, Johnson EG, Hijaz FM, Killiny N, Hilf ME (2014). Viability of ‘*Candidatus* Liberibacter asiaticus’ prolonged by addition of citrus juice to culture medium. Phytopathology..

[65] Ha PT, He R, Killiny N, Brown JK, Omsland A, Gang DR (2019). Host-free biofilm culture of “*Candidatus* Liberibacter asiaticus,” the bacterium associated with Huanglongbing. Biofilm..

[66] Achor D, Etxeberria E, Wang N, Folimonova S, Chung K, Albrigo L (2010). Citrus affected with huanglongbing disease. Plant Pathol J.

[67] Koh E-J, Zhou L, Williams DS, Park J, Ding N, Duan Y-P (2012). Callose deposition in the phloem plasmodesmata and inhibition of phloem transport in citrus leaves infected with “*Candidatus* Liberibacter asiaticus”. Protoplasma..

