# Supplementary figures and images for: Salicylic acid mediated immune response of Citrus sinensis to varying frequencies of herbivory and pathogen inoculation
Source: BMC Plant Biol. 2022 Jan 3;22:7. doi: 10.1186/s12870-021-03389-5 (PMC8722004; doi:10.1186/s12870-021-03389-5)

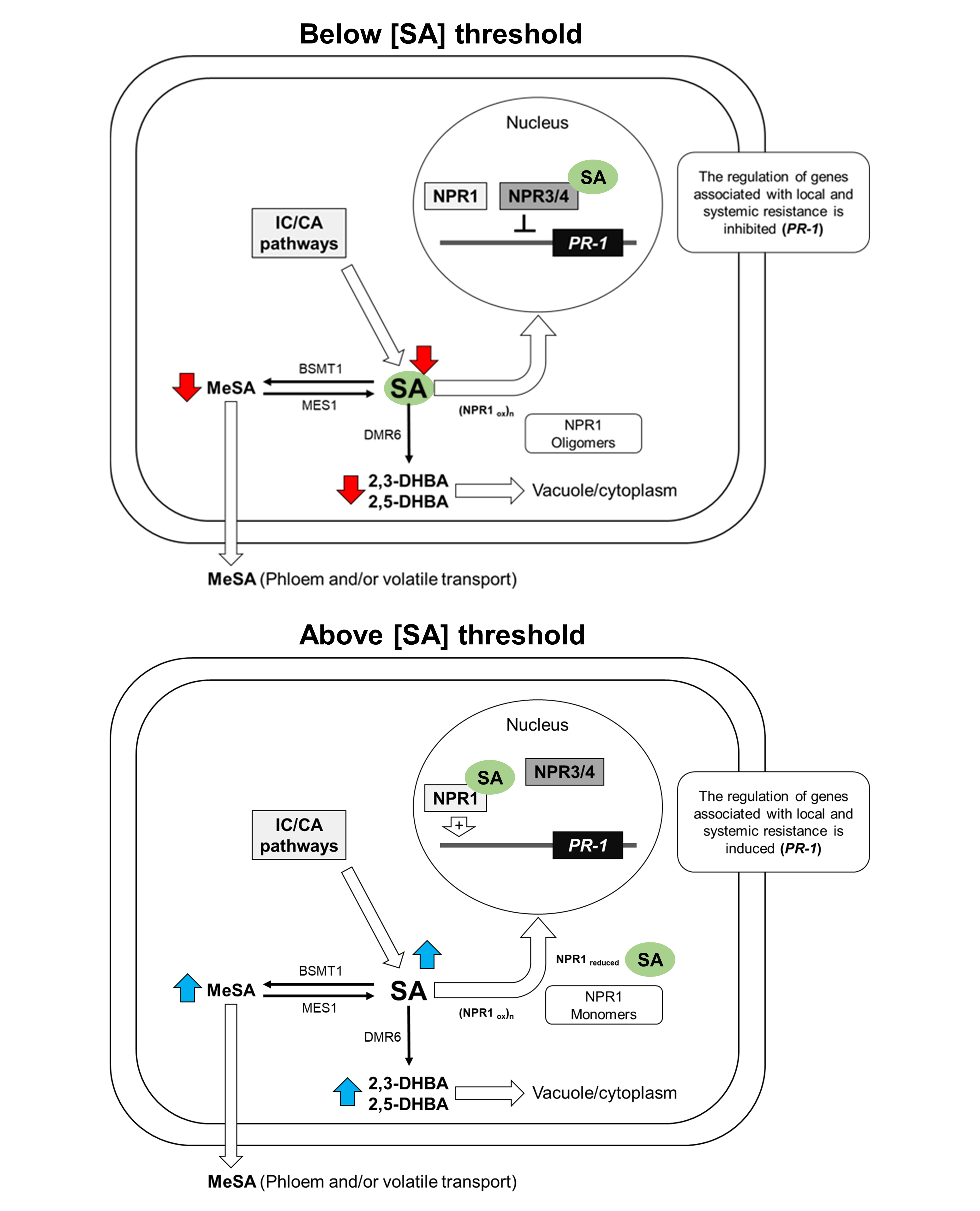

Supplement: Supplementary file 1 — Additional file 1: S1 Fig. Proposed models of SA-dependent immune responses in C. sinensis. The figure was created and designed by the authors. [file 12870_2021_3389_MOESM1_ESM.jpg]
